# Dynamic Changes and Nomogram Prediction for Sinistral Portal Hypertension in Moderate and Severe Acute Pancreatitis

**DOI:** 10.3389/fmed.2022.875263

**Published:** 2022-05-26

**Authors:** Chen Yu, Ling Ding, Meilan Jiang, Qian Liao, Xin Huang, Yupeng Lei, Huajing Ke, Huifang Xiong, Wenhua He, Liang Xia, Xianjun Zeng, Nonghua Lu, Yin Zhu

**Affiliations:** ^1^Department of Gastroenterology, The First Affiliated Hospital of Nanchang University, Nanchang, China; ^2^Jiangxi Province Medical Imaging Research Institute, Nanchang, China; ^3^Gaoxin Hospital of the First Affiliated Hospital of Nanchang University, Nanchang, China; ^4^Department of Radiology, The First Affiliated Hospital of Nanchang University, Nanchang, China

**Keywords:** acute pancreatitis, sinistral portal hypertension, nomogram, severe acute pancreatitis, prediction

## Abstract

**Objectives:**

To investigate the dynamic changes in gastric varices in patients with acute pancreatitis (AP) and to develop a novel nomogram for the early prediction of sinistral portal hypertension (SPH).

**Methods:**

This was a retrospective, case-control study with an analysis of the quantitative, dynamic computed tomography imaging results of SPH in patients with moderate and severe AP with a long-term follow-up. Multivariate logistic regression analysis and nomogram were employed.

**Results:**

The SPH group (*n* = 94) and non-SPH group (*n* = 94) were matched. The dynamic changes showed an increasing trend in the diameter of gastric fundus, short gastric, gastric coronary, and gastroepiploic veins, which did not recover during the one-year follow-up. Multivariate analysis showed that male (adjusted odds ratio (adjOR), 8.71; 95% confidence interval (CI), 2.86–26.53; *P* < 0.001), body mass index ≥27.5 kg/m^2^ (adjOR, 5.49; 95% CI, 1.85–16.29; *P* = 0.002), prothrombin time ≥12.6 s (adjOR, 2.82; 95% CI, 1.11–7.17; *P* = 0.03), and the patency of splenic vein [stenosis (adjOR, 8.48; 95% CI, 2.13–33.71; *P* = 0.002), and occlusion (adjOR, 34.57; 95% CI, 10.87–110.00; *P* < 0.001)] were independently associated with the development of SPH. The nomogram incorporating these factors demonstrated good discrimination, calibration and clinical utility. The area under the curve was as high as 0.92 (95% CI, 0.87–0.95).

**Conclusion:**

The dynamic changes in varices in SPH are long-term and slowly progressing. Males and obese patients with abnormal splenic veins and coagulopathies are at high risk for developing SPH. A simple nomogram tool helps in the early, accurate prediction of SPH.

## Introduction

Sinistral portal hypertension (SPH) is a localized form of portal hypertension that usually occurs as a result of isolated obstruction of the splenic vein (1). Since the splenic vein is contiguous with the pancreas, pancreatic disorders, include pancreatitis and pancreatic neoplasms, are the most common etiology (2). Because most patients with SPH in acute pancreatitis (AP) are asymptomatic, in the past, SPH has been easily ignored clinically (3). However, due to the increased awareness of the entity and recent advances in the diagnostic approaches, especially the wide application of computed tomography (CT) for assessing pancreatic pathology, SPH is often found incidentally on investigation (3). SPH was detected in 3.3% of AP patients and in 12.5% of patients with moderate and severe AP (3, 4). For patients without any symptoms, correct prognostic judgment and advice on the follow-up are important for the medical consultation. In addition, very few patients with SPH run the risk for gastrointestinal bleeding from gastric varices, which is usually serious and potentially fatal (5–7). Thus, understanding of the diagnosis, risk stratification, and prognosis of SPH imposes important clinical significance, and this understanding may help with developing suitable discharge plan and improving outpatient management.

Splenic venous occlusion secondary to thrombosis formation, inflammatory stenosis, or external compression results in venous hypertension in collateral pathways that carry the splenic blood to the superior mesenteric and portal veins, mainly including the short gastric, coronary, and gastroepiploic veins (8). Xie et al. (3) reported 633 AP patients, including 21 patients diagnosed with SPH, and found that isolated gastric varices detected included gastroepiploic varices (100%), short gastric varices (61.9%), coronary varices (42.9%), and fundal varices (19.0%). However, the dynamic changes in varices have not been reported, which is critical for SPH due to its complex, prolonged clinical course. In addition, few studies have focused on the risk factors for SPH in patients with AP. Li et al. (4) demonstrated that males, recurrent AP, hypertriglyceridemia, high glucose, and infection of walled-off pancreatic necrosis were independent risk factors for SPH in patients with moderate and severe AP. However, the predictive value of these factors was not assessed, which made it difficult to derive consistent clinical guidance. There is potential significant benefit in using the clinical and imaging indicators determined at early stage of disease to predict SPH as early as possible, but this type of data has rarely been reported. A weighted risk score that can assist physicians in distinguishing high-risk patients from low-risk patients with SPH is still urgently needed clinically.

Thus, in this case-control study, we aimed to (1) investigate the imaging features and dynamic changes of gastric varices in patients with AP on contrast-enhanced CT; (2) analyze the relationship between the patency of portosplenomesenteric veins and the development of SPH; and (3) clarify the independent risk factors and develop a novel nomogram for the early prediction of SPH.

## Materials and Methods

### Study Design and Participants

This was a retrospective, case-control study that analyzed AP patients who were hospitalized in the Department of Gastroenterology of the First Affiliated Hospital of Nanchang University, a tertiary care referral center in China, from January 2015 to October 2019. We used data from a prospectively maintained database, which is a data repository for the clinical data of all AP patients admitted to our department, and this data included diagnostic, therapeutic, and follow-up data that was recorded by a special research assistant. Patients with moderate severe AP (MSAP) and severe AP (SAP) diagnosed according to the 2012 Atlanta Classification criteria were eligible (9). Patients who were admitted to the hospital 2 weeks after onset of AP were excluded due to lack of available images taken at early stage of the disease. Patients complicated with chronic pancreatitis, pancreatic cancer, chronic liver disease, cirrhosis, peritoneal/retroperitoneal tumors, or history of gastric, splenic or pancreatic surgery were also excluded due to the confounding effect on the evaluation of the varices. All patients were followed up for at least 1 year after discharge, and the patients who were lost to follow-up were excluded. A total of 316 patients were eligible. Among them, 94 patients were diagnosed with SPH (SPH group). Among the 222 patients without SPH, 94 patients were randomly matched on age (non-SPH group) using SPSS software (v20.0; SPSS Inc., Chicago, IL, United States). The SPH group and 1:1 age-matched non-SPH group were compared. The AP database was approved by the Ethics Committee of the First Affiliated Hospital of Nanchang University (N0: 2011001). Written informed consent was waived due to the retrospective nature of the study.

### Image Analysis

Contrast-enhanced CT imaging was obtained within 2 weeks after the onset of AP in all of the patients analyzed to determine the early imaging features for early prediction. Additionally, Contrast-enhanced CT or CT venography examinations were performed at follow-up to analyze the dynamic changes in imaging features. All of the images were reassessed and reviewed by two senior radiologists specializing in abdominal imaging who had more than 10 years of experience and were blinded to the clinical data and outcome parameters. All CT examinations were performed using a 128-detector CT scanner (Somatom Definition AS +, Siemens Medical Systems, Erlangen, Germany) or a 256-detector CT scanner (Revolution CT, GE Healthcare, Milwaukee, WI, United States). 1.3 mL/kg iopromide (Ultravist 370; Bayer Schering Pharma, Berlin, Germany) was intravenously injected at a rate of 3 mL/s with a high-pressure injector. Then, when the attenuation of the aorta at the thoracolumbar junction had reached 180 HU and a fixed 60-s delay, the contrasting arterial and portal phase scans were obtained separately. The scanned area extended from the diaphragmatic domes to the pubic symphysis inferior pole of right kidney depending on clinical needs. The scan parameters were as follows: a detector collimation of 64 × 0.625 mm, a beam pitch of 0.984, a kVp of 120, an automated dose modulation using a maximum allowable tube current set at 200 mAs, and a section thickness/reconstruction interval of 2 mm/2 mm (10).

### Data Collection and Definition

**FIGURE 1:**
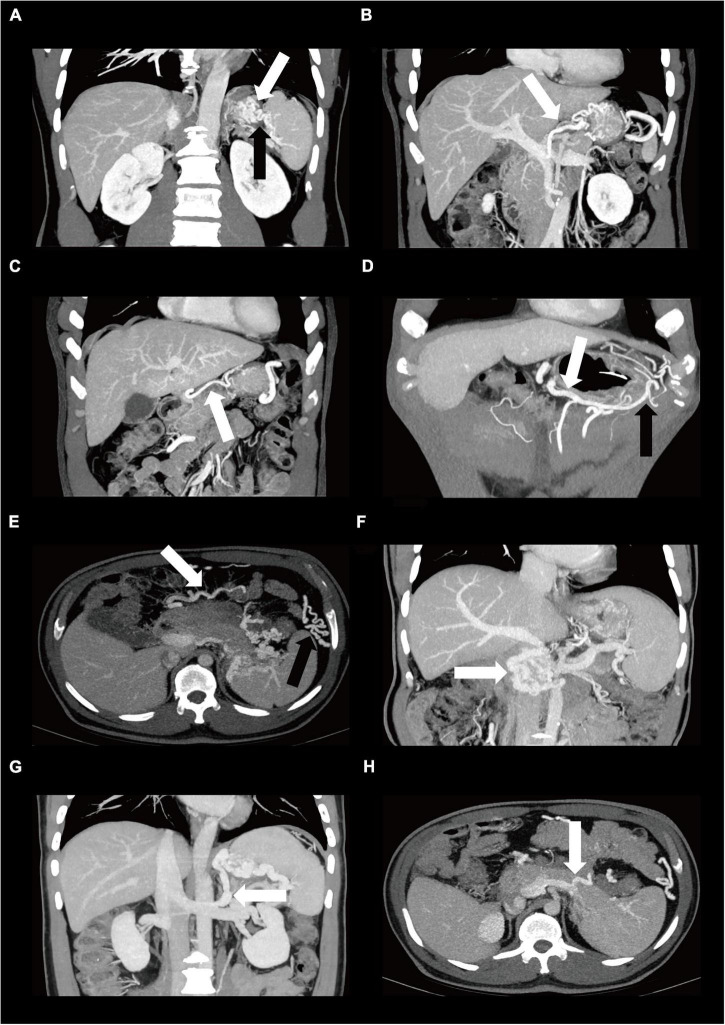
The Contrast-enhanced CT images of varices in patients diagnosed as SPH. One of the main collateral pathway is through enlarged short gastric varices (**A**, black arrow) and gastric fundus varices (**A**, white arrow) *via* gastric coronary varices (**B**, white arrow) or rarely *via* right gastric varices (**C**, white arrow) drained into the portal vein. Another collateral pathway is through left gastroepiploic varices (**D**, black arrow) *via* right gastroepiploic varices (**D**, white arrow) drained into the superior mesenteric vein. Other abnormal varices due to portal hypertension include middle colonic vein (**E**, white arrow), omentum branch of the gastroepiploic vein (**E**, black arrow), pancreaticoduodenal vein (**F**, white arrow), left adrenal vein (**G**, white arrow), and dorsal pancreatic vein (**H**, white arrow). CT, computed tomography, SPH, sinistral portal hypertension.

Patients with AP who had no signs of liver disease but had isolated gastric varices and/or splenomegaly were considered to have SPH (3, 11). The presence of collaterals vessels was defined as disproportionate increases in the caliber and in the number of vessels in any location. However, not all gastric varices were a result of portal hypertension. The condition in which the proximal vein is not enlarged and the distal vein in the collateral pathways is enlarged may be caused by the reflux in other vessels. Thus, in our study, varices were diagnosed when both short gastric vein and gastric coronary vein were enlarged in the collateral pathway drained into the portal vein or if both left gastroepiploic vein and right gastroepiploic vein were enlarged in the collateral pathway drained into the superior mesenteric vein. Varices were indicated when the maximum diameter was greater than 5 mm for short gastric vein, 6 mm for gastric coronary vein, 6 mm for gastroepiploic vein, and 4 mm for middle colonic vein (3). Also, when gastric fundus vein was located in the gastric fundal wall, varices were indicated (3). In this paper, we unexpectedly found some rare abnormal varices due to portal hypertension involving right gastric vein, omentum branch of the gastroepiploic vein, pancreaticoduodenal vein, left adrenal vein, and dorsal pancreatic vein. The CT images of these involved varices are shown in Figure 1.

The portal venous system, including portal splenic, and superior mesenteric veins, was evaluated for patency of vessel (normal, stenosis, occlusion), and occurrence of thrombosis (12). Occlusion of a vein was defined as complete occlusion without blood flow of the vessel. Stenosis of a vein was defined as a more than a 50% decrease in the caliber of the lumen. Otherwise, it was defined as a “normal” vein. Thrombosis was defined as an intravenous filling defect in the lumen on contrast-enhanced CT. Other imaging features, including pancreatic parenchyma and local complications, were also recorded to explore their value for prediction. A non-enhancing area of pancreatic parenchyma should be considered to be pancreatic parenchymal necrosis on contrast-enhanced CT (9). In peripancreatic necrosis, the pancreas enhances normally on contrast-enhanced CT, but the peripancreatic tissues develop necrosis (9). Local complications included acute peripancreatic fluid collection (APFC), and acute necrotic collection (ANC). APFC was a peripancreatic fluid associated with edematous pancreatitis (9). ANC was defined as a collection containing variable amounts of both fluid and necrosis associated with necrotizing pancreatitis (9). Other detailed records of baseline characteristics, serum indicators within 24 h of admission, and clinical outcomes were also collected from the AP database and compared between patients with SPH and without SPH.

### Statistical Analysis

Quantitative variables are presented as medians (interquartile ranges, IQRs) and were analyzed using the signed-rank test. Categorical variables are reported as absolute numbers and proportions and were tested by McNemar’s test. Factors associated with the occurrence of SPH in unadjusted models (*P* < 0.1) were included in the multivariate logistic regression analysis (forward stepwise) to identify the independent risk factors. A nomogram was constructed on the basis of the outcome of the final multivariable regression analysis. Receiver operating characteristic (ROC) curves were plotted to assess the discrimination ability of the model. Calibration, which is the level of agreement between the predicted and actual risks, was evaluated by the Hosmer-Lemeshow χ2 test and calibration curve. Decision curve analysis (DCA) was performed to assess the clinical usefulness of the model by quantifying the net benefits under different threshold probabilities. A two-tailed *P*-value <0.05 was considered statistically significant. Data were analyzed using SPSS software (v20.0; SPSS Inc., Chicago, IL, United States) or R software (version 4.0.5, R Development Core Team).

## Results

### Features and Dynamic Changes of Gastric Varices

**FIGURE 2:**
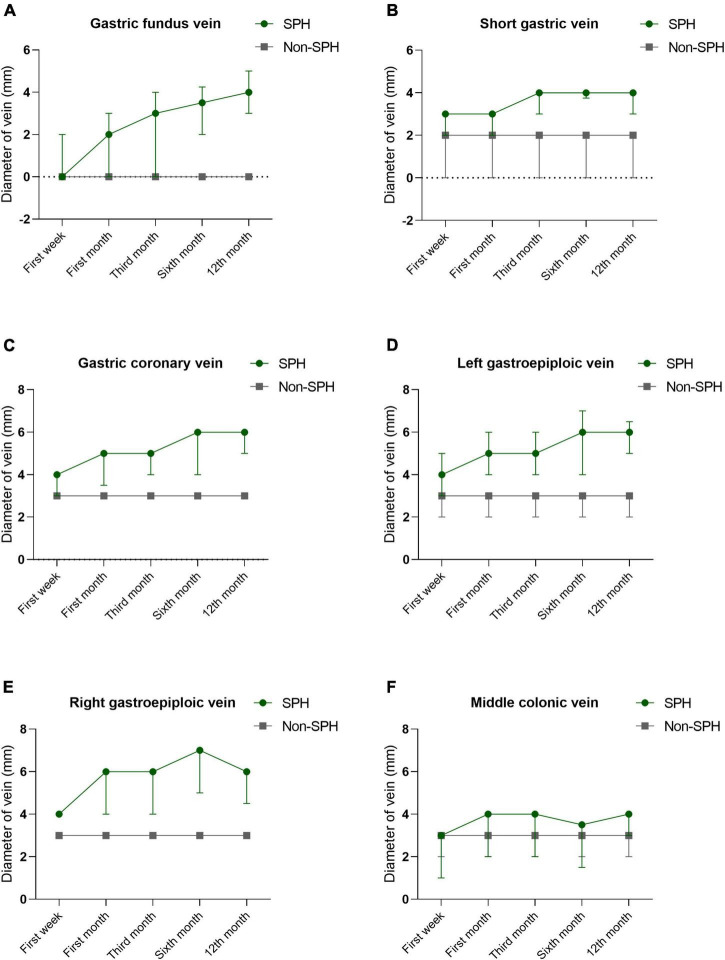
The Dynamic changes of maximum diameters in varicosed veins during the first week, first month, third month, sixth month, and 12th month of follow-up. Most common gastric varices analyzed include gastric fundus vein **(A)**, short gastric vein **(B)**, gastric coronary vein **(C)**, left gastroepiploic vein **(D)**, right gastroepiploic vein **(E)**, and middle colonic vein **(F)**, and it shows an increasing trend in the diameter for these varices, which did not recover within 1 year.

A total of 188 MSAP and SAP patients were included in the study, and the mean follow-up time was 588 ± 329 days, ranging from 365 to 1884 days. The SPH group (*n* = 94) and non-SPH group (*n* = 94) were 1:1 matched for age. The most detected isolated gastric varices were gastric fundus varices (90.4%, 85/94), followed by right gastroepiploic varices (75.5%, 71/94), left gastroepiploic varices (70.2%, 66/94), middle colonic vein (68.1%, 64/94), gastric coronary varices (61.7%, 58/94), short gastric varices (57.4%, 54/94), and omentum branch of the gastroepiploic varices (53.2%, 50/94). We also observed some rarely reported varicosed veins due to the development of portal hypertension, and these veins included pancreaticoduodenal vein (*n* = 8), right gastric vein (*n* = 1), left adrenal vein (*n* = 2), and dorsal pancreatic vein (*n* = 1). The CT images of these gastric varices are shown in Figure 1. The diameters of varicosed veins, including gastric fundus, short gastric, gastric coronary, left gastroepiploic, and right gastroepiploic veins, were significantly wider in patients with SPH than in controls (*P* < 0.001) during the first week, first month, third month, sixth month, and 12th month of follow-up. The dynamic changes showed an increasing trend in the diameter for these varices, which did not recover within 1 year. The details of the dynamic changes in varices are outlined in Figure 2 and Supplementary Table 1. The median (IQR) time until the first detection of SPH was 9 (4, 36) weeks, ranging from 1 to 225 weeks. SPH was diagnosed in 36 patients (38.3%) within 1 month after AP onset, in 52 patients (55.3%) between 1 month and 1 year, and in 6 patients (6.4%) after more than 1 year.

### Baseline Characteristics, Clinical Profile, and Imaging Features

The proportion of males was significantly higher in the patients with SPH than in those without SPH (88.3% vs. 54.3%; *P* < 0.001), and the median age of all the patients was 44.0 (IQR, 36.0–52.0) years. More smokers (30.9% vs. 13.8%; *P* = 0.005) and drinkers (37.2% vs. 22.3%; *P* = 0.026) were observed in the SPH group than in the control group. There were no significant differences in terms of age, etiologies, history of AP, hypertension, diabetes, or time from AP onset to admission. The serum laboratory indicators within 24 h of admission were also compared, and differences were found in terms of renal function and coagulation indicators. The serum levels of blood urea nitrogen (6.0 (4.5, 10.7) mmol/L vs. 5.3 (4.0, 7.3) mmol/L; *P* = 0.005), creatinine (68.6 (57.2, 103.5) μmol/L vs. 58.9 (46.9, 78.0) μmol/L; *P* = 0.001), prothrombin time (PT) (12.8 (11.8, 13.9) seconds vs. 12.0 (11.1, 12.7) seconds; *P* < 0.001), and international normalized ratio (1.2 (1.1, 1.2) vs. 1.1 (1.0, 1.2); *P* = 0.041) were significantly increased in patients with SPH compared to those without SPH. The detailed baseline characteristics, serum indictors, and clinical outcomes are depicted in Table 1. Patients with SPH were combined with more pancreatic parenchyma necrosis (70.2% vs. 28.8%; *P* < 0.001), and necrosis was mainly located in neck (30.9% vs. 17.0%; *P* = 0.026) and body/tail (61.7% vs. 16.0%; *P* < 0.001) of pancreas. More information about the imaging features of pancreatic parenchyma and local complications is outlined in Table 2.

**TABLE 1:** Baseline characteristics, serum laboratory indicators, and clinical outcomes of all patients.

	All (*n* = 188)	SPH group (*n* = 94)	Non-SPH group (*n* = 94)	*P*-value
**Baseline characteristics**				
**Sex**				**<0.001**
Male	134 (71.3%)	83 (88.3%)	51 (54.3%)	
Female	54 (28.7%)	11 (11.7%)	43 (45.7%)	
Age, yr	44.0 (36.0, 52.0)	44.0 (35.0, 52.0)	44.0 (36.0, 52.0)	0.848
BMI, kg/m^2^	24.2 (22.2, 27.2)	24.5 (22.2, 27.9)	23.7 (21.9, 26.4)	0.148
**Etiology**				**0.169**
Biliary	61 (32.4%)	25 (26.6%)	36 (38.3%)	
Hyperlipidemia	74 (39.4%)	38 (40.4%)	36 (38.3%)	
Alcoholic	18 (9.6%)	9 (9.6%)	9 (9.6%)	
Mixed	15 (8.0%)	11 (11.7%)	4 (4.3%)	
Others	20 (10.6%)	11 (11.7%)	9 (9.6%)	
History of AP	44 (23.4%)	23 (24.5%)	21 (22.3%)	0.730
History of hypertension	21 (11.2%)	12 (12.8%)	9 (9.6%)	0.487
History of diabetes	27 (14.4%)	16 (17.0%)	11 (11.7%)	0.298
Smoking status	42 (22.3%)	29 (30.9%)	13 (13.8%)	**0.005**
Drinking status	56 (29.8%)	35 (37.2%)	21 (22.3%)	**0.026**
Time from AP onset to admission, days	3.0 (2.0, 7.0)	3.0 (2.0, 8.3)	3.0 (2.0, 6.0)	0.169
**Serum indicators within 24 h of admission**				
CRP, mg/L	191.0 (110.0, 305.0)	214.0 (81.1, 342.8)	184.0 (115.5, 274.0)	0.416
PCT, ng/ml	1.2 (0.3, 4.1)	1.3 (0.4, 5.5)	1.2 (0.3, 3.0)	0.472
HCT, L/L	37.1 (32.3, 42.0)	36.4 (32.4, 41.2)	38.9 (32.1, 42.9)	0.245
BUN, mmol/L	5.5 (4.1, 9.0)	6.0 (4.5, 10.7)	5.3 (4.0, 7.3)	**0.005**
Cr, umol/L	64.4 (51.3, 83.3)	68.6 (57.2, 103.5)	58.9 (46.9, 78.0)	**0.001**
PT, seconds	12.2 (11.5, 13.2)	12.8 (11.8, 13.9)	12.0 (11.1, 12.7)	**<0.001**
APTT, seconds	30.3 (26.2, 36.0)	30.3 (26.2, 35.1)	30.3 (25.9, 36.3)	0.608
D-dimer, mg/L FEU	4.6 (2.4, 8.3)	4.4 (2.6, 7.9)	5.0 (2.3, 9.0)	0.514
INR	1.1 (1.1, 1.2)	1.2 (1.1, 1.2)	1.1 (1.0, 1.2)	**0.041**
Fibrinogen, g/L	5.5 (4.4, 6.7)	5.6 (4.3, 6.8)	5.4 (4.4, 6.7)	0.673
TT, seconds	16.9 (15.0, 18.9)	17.1 (15.6, 18.3)	16.4 (14.4, 19.2)	0.335
**Clinical outcomes**				
Use of LMWH	65 (34.6%)	36 (38.3%)	29 (30.9%)	0.283
Severity of AP				0.078
MSAP	106 (56.4%)	47 (50.0%)	59 (62.8%)	
SAP	82 (43.6%)	47 (50.0%)	35 (37.2%)	
*Persistent respiratory failure	77 (41.0%)	43 (45.7%)	34 (36.2%)	0.182
*Persistent renal failure	17 (9.0%)	14 (14.9%)	3 (3.2%)	**0.005**
*Persistent circulatory failure	12 (6.4%)	8 (8.5%)	4 (4.3%)	0.233
Sepsis	21 (11.2%)	17 (18.1%)	4 (4.3%)	**0.003**
IPN	48 (25.5%)	32 (34.0%)	16 (17.0%)	**0.007**
Length of hospital stay, days	17.0 (12.0, 29.8)	19.5 (13.0, 35.3)	16.0 (11.0, 28.0)	0.097

Date are median (interquartile ranges) or absolute numbers (proportions). SPH, sinistral portal hypertension; BMI, body mass index; AP, acute pancreatitis; CRP, C-reactive protein; PCT, procalcitonin; HCT, hematocrit; BUN, blood urea nitrogen; Cr, creatinine; PT, prothrombin time; APTT, activated partial thromboplastin time; INR, international normalized ratio; TT, thrombin time; LMWH, low molecular weight heparin; MSAP, moderately severe acute pancreatitis; SAP, severe acute pancreatitis; IPN, infected pancreatic necrosis. *Persistent organ failure were assessed according to the 2012 Atlanta Classification of AP. P < 0.05 was bolded.

**TABLE 2:** Imaging features on CECT within 2 weeks of AP onset.

	All (*n* = 188)	SPH group (*n* = 94)	Non-SPH group (*n* = 94)	*P*-value
**Portosplenomesenteric vein**				
Portal vein				**0.003**
Normal	177 (94.1%)	83 (88.3%)	94 (100.0%)	
Stenosis	10 (5.3%)	10 (10.6%)	0 (0.0%)	
Occlusion	1 (0.5%)	1 (0.5%)	0 (0.0%)	
Portal vein thrombosis	6 (3.2%)	6 (6.4%)	0 (0.0%)	**0.029**
Splenic vein				**<0.001**
Normal	105 (55.9%)	23 (24.5%)	82 (87.2%)	
Stenosis	22 (11.7%)	17 (18.1%)	5 (5.3%)	
Occlusion	61 (32.4%)	54 (57.4%)	7 (7.4%)	
Splenic vein thrombosis	24 (12.8%)	22 (23.4%)	2 (2.1%)	**<0.001**
SMV				**0.029**
Normal	182 (96.8%)	88 (93.6%)	94 (100.0%)	
Stenosis	6 (3.2%)	6 (6.4%)	0 (0.0%)	
SMV thrombosis	5 (2.7%)	5 (5.3%)	0 (0.0%)	0.059
**Pancreatic parenchyma**				
Type of pancreatic necrosis				**<0.001**
Non-necrosis	29 (15.4%)	14 (14.9%)	15 (16.0%)	
EXPN only	66 (35.1%)	14 (14.9%)	52 (55.3%)	
PPN only	7 (3.7%)	3 (3.2%)	4 (4.3%)	
PPN and EXPN	86 (45.7%)	63 (67.0%)	23 (24.5%)	
Proportion of PPN,%				**<0.001**
0	97 (51.6%)	29 (30.9%)	68 (72.3%)	
<30	39 (20.7%)	22 (23.4%)	17 (18.1%)	
30–50	21 (11.2%)	18 (19.1%)	3 (3.2%)	
>50	31 (16.5%)	25 (26.6%)	6 (6.4%)	
**Location of PPN**				
Head	31 (16.5%)	19 (20.2%)	12 (12.8%)	0.169
Neck	45 (23.9%)	29 (30.9%)	16 (17.0%)	**0.026**
Body-tail	73 (38.8%)	58 (61.7%)	15 (16.0%)	**<0.001**
**Local complications**				
Fluid collection				0.231
Non-collection	35 (18.6%)	19 (20.2%)	16 (17.0%)	
APFC	6 (3.2%)	1 (1.1%)	5 (5.3%)	
ANC	147 (78.2%)	74 (78.7%)	73 (77.7%)	
Number of fluid collection	3 (1, 5)	3 (1, 5)	3 (1, 5)	0.553
**Location of fluid collection**				
Transverse mesocolon	126 (67.0%)	62 (66.0%)	64 (68.1%)	0.756
Left anterior renal space	130 (69.1%)	65 (69.1%)	65 (69.1%)	−
Right anterior renal space	76 (40.4%)	31 (33.0%)	45 (47.9%)	**0.037**
Left posterior renal space	37 (19.7%)	17 (18.1%)	20 (21.3%)	0.582
Right posterior renal space	21 (11.2%)	7 (7.4%)	14 (14.9%)	0.105
Left perirenal space	46 (24.5%)	23 (24.5%)	23 (24.5%)	−
Right perirenal space	29 (15.4%)	11 (11.7%)	18 (19.1%)	0.158
Mesenteric root	11 (5.9%)	5 (5.3%)	6 (6.4%)	0.756
Pelvic cavity	3 (1.6%)	1 (1.1%)	2 (2.1%)	−
Greater omentum	28 (14.9%)	12 (12.8%)	16 (17.0%)	0.413
Lesser omentum	67 (35.6%)	33 (35.1%)	34 (36.2%)	0.879
Left iliac fossa	39 (20.7%)	23 (24.5%)	16 (17.0%)	0.208
Right iliac fossa	14 (7.4%)	6 (6.4%)	8 (8.5%)	0.578
Presacral space	5 (2.7%)	2 (2.1%)	3 (3.2%)	−
Left paracolic sulci	2 (1.1%)	2 (2.1%)	0 (0.0%)	0.497
Right paracolic sulci	1 (0.5%)	1 (1.1%)	0 (0.0%)	−

*Date are median (interquartile ranges) or absolute numbers (proportions). CECT, contrast-enhanced computed tomography; AP, acute pancreatitis; SPH, sinistral portal hypertension; SMV, superior mesenteric vein; EXPN, extrapancreatic necrosis; PPN, pancreatic parenchymal necrosis; APFC, acute peripancreatic fluid collection; ANC, acute necrotic collection. P < 0.05 was bolded.*

### Portosplenomesenteric Veins and Their Association With Sinistral Portal Hypertension

**FIGURE 3:**
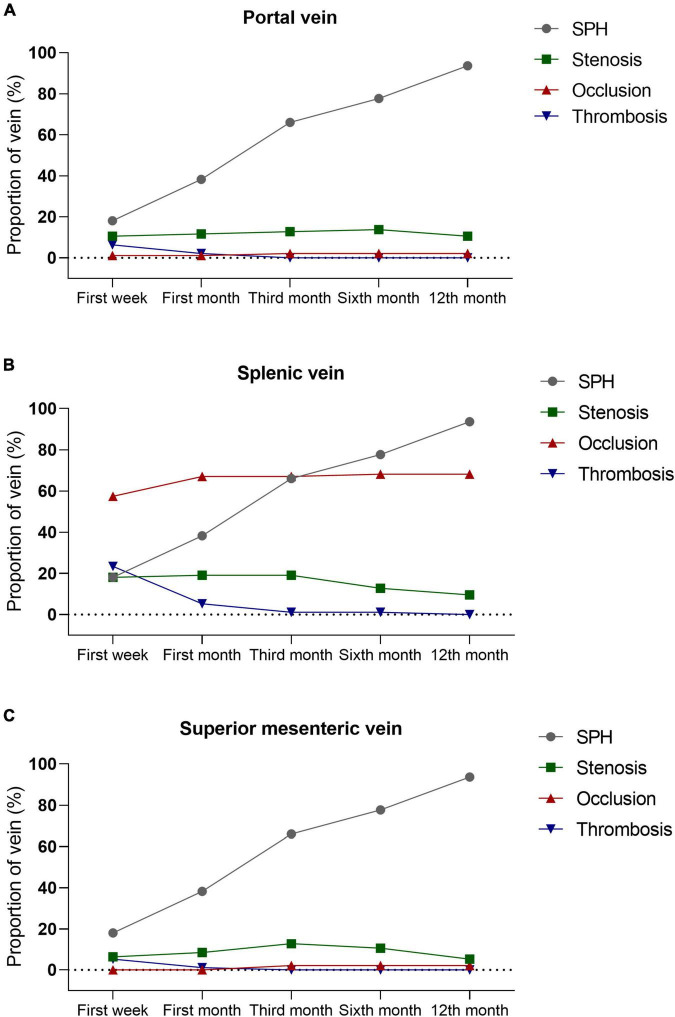
The Patency of portosplenomesenteric veins and their association with SPH. Thrombosis of portal vein **(A)**, splenic vein **(B)**, and superior mesenteric vein **(C)** are usually detected within 1 month after the onset of AP and undetected 3 months after the onset of AP. Unlike thrombosis, stenosis and occlusion of portosplenomesenteric vein tend to appear at the early stage of disease and persist during the one-year follow-up period. SPH, sinistral portal hypertension; AP, acute pancreatitis.

Sinistral portal hypertension was strongly associated with the patency of portosplenomesenteric veins, with splenic vein being the most common abnormal vein, followed by portal and superior mesenteric veins. Patients with SPH were complicated with more occlusion (57.4% vs. 7.4%) and stenosis (18.1% vs. 5.3%) of splenic vein (*P* < 0.001), as well as splenic venous thrombosis (23.4% vs. 2.1%; *P* < 0.001). Abnormalities were also detected in portal vein (stenosis, 10.6%; occlusion, 0.5%; thrombosis, 6.4%) and superior mesenteric vein (stenosis, 6.4%; thrombosis, 5.3%) in SPH patients, but there were no abnormalities of these veins in the control group. In the SPH group, the proportion of patients diagnosed with SPH increased over time [first week, 17 (18.1%); first month, 36 (38.3%); third month, 62 (66.0%); sixth month, 73 (77.7%); 12th month, 88 (93.8%)]. However, the dynamic changes in the patency of portosplenomesenteric veins were different from those of SPH. Mostly, thrombosis of splenic vein was detected within 1 month after the onset of AP and undetected 3 months after the onset of AP. Unlike thrombosis, stenosis and occlusion of splenic vein tended to appear at an early stage of the disease and persist during the one-year follow-up period. Similar dynamic changes were also observed in portal and superior mesenteric veins. The dynamic changes in the patency of portosplenomesenteric veins are shown in Figure 3 and Supplementary Table 2. In addition to the portosplenomesenteric vein, SPH also could be related to other rarely affected veins. For example, Varicose pancreaticoduodenal vein in 8 patients in our study were related to stenosis or occlusion of the inferior mesenteric vein.

### Predictive Factors and Nomogram Model for Sinistral Portal Hypertension

**FIGURE 4:**
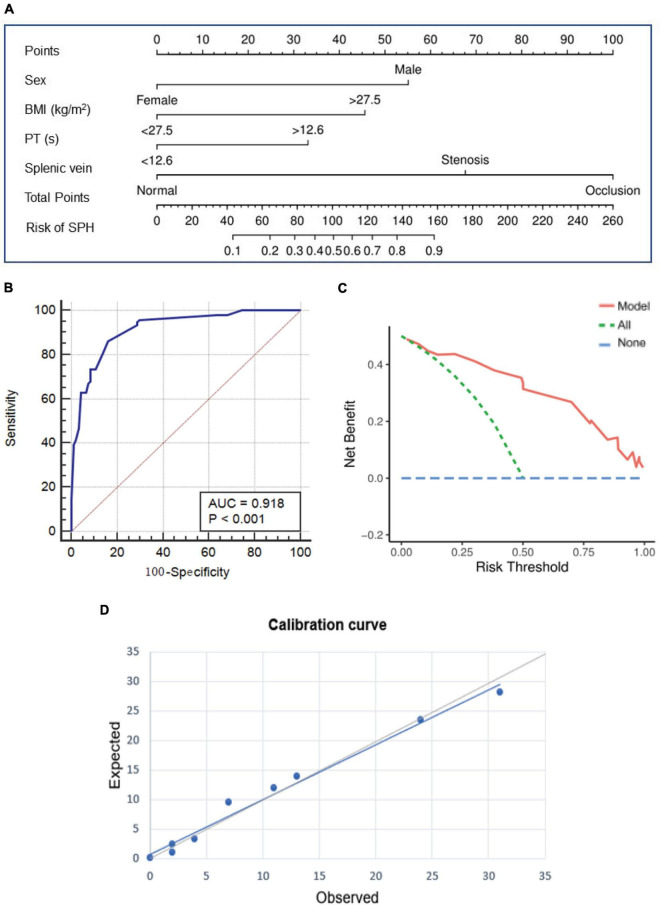
The prediction nomogram and its performance for SPH. **(A)** Nomogram based on multivariate Logistic regression analysis is developed for the prediction of SPH. ROC curve **(B)** decision curve analysis **(C)**, and calibration curve **(D)** show the good discrimination, calibration, and clinical utility of the novel model. SPH, sinistral portal hypertension; ROC, receiver operating characteristics.

The results of univariate analysis are detailed in Supplementary Table 3. The factors associated with SPH in the unadjusted models (*P* < 0.1) were included in the multivariable models. After adjusting for the baseline characteristics, serum indicators at admission and imaging features, multivariate analysis showed that male sex (adjusted odds ratio (adjOR), 8.71; 95% confidence interval (CI), 2.86–26.53; *P* < 0.001), body mass index (BMI) ≥27.5 kg/m^2^ (adjOR, 5.49; 95% CI, 1.85–16.29; *P* = 0.002), PT ≥12.6 s (adjOR, 2.82; 95% CI, 1.11–7.17; *P* = 0.03), the patency of splenic vein (stenosis (adjOR, 8.48; 95% CI, 2.13–33.71; *P* = 0.002), and occlusion [adjOR, 34.57; 95% CI, 10.87–110.00; *P* < 0.001)] were independently associated with the development of SPH (Table 3). Based on the multivariate analysis, a novel nomogram was constructed by assigning a weighted point value to each independent risk factor on the scale (Figure 4A). A higher total score for all of the risk factors was associated with a higher risk of SPH. The discriminatory ability of the prediction model as measured by the area under the curve (AUC) was as high as 0.92 (95% CI, 0.87–0.95), with a sensitivity of 86.17% and a specificity of 84.04% (Figure 4B). As shown in Figure 4C, DCA analysis implied that the nomogram was clinically useful for the prediction of reintervention. The novel model was not significantly different from a perfect fit (χ^2^ = 6.25, *P* = 0.51), and the calibration curve also showed good calibration (Figure 4D).

**TABLE 3:** Multivariate Logistic regression analysis for SPH in patients with AP.

	B	Adjusted OR (95%CI)	*P*-value
**Sex (ref: female)**			
Male	2.16	8.71 (2.86, 26.53)	**<0.001**
**BMI (ref: <27.5 kg/m^2^)**			
≥27.5	1.70	5.49 (1.85, 16.29)	**0.002**
**PT (ref: <12.6 s)**			
≥12.6	1.04	2.82 (1.11, 7.17)	**0.030**
**Splenic vein (ref: normal)**			
Stenosis	2.14	8.48 (2.13, 33.71)	**0.002**
Occlusion	3.54	34.57 (10.87, 110.00)	**<0.001**

*Factors associated with the occurrence of SPH in unadjusted models (P < 0.1) were included in the multivariable models, and only results with P < 0.05 in the multivariate regression were listed above. Receiver operating characteristics (ROC) curves were constructed to determine the optimal threshold for predicting clinical outcomes. SPH, sinistral portal hypertension; AP, acute pancreatitis; OR, odds ratio; CI, confidence interval; BMI, body mass index; PT, prothrombin time. P < 0.05 was bolded.*

## Discussion

SPH is a common, prolonged complication in patients with moderate and severe AP, and it is associated with high incidence and heavy medical burden (4). Studies on dynamic changes in gastric varices and prognostic models in SPH have rarely been reported. In this observational study based on contrast-enhanced CT imaging using a long-term follow-up, we clarified that the development of gastric varices in SPH patients was a long-term, slowly progressing disease course. The patency of splenic vein was strongly associated with the development of SPH, with persistent occlusion and stenosis of splenic vein being the main factors. Male sex, high BMI, prolonged PT, and abnormal splenic vein were independent risk factors for SPH. The nomogram based on these risk factors could greatly predict SPH with good discrimination, calibration and clinical utility.

Normal liver condition, isolated gastric varices, and splenomegaly are characteristics of SPH, among which the radiological evaluation of gastric varices plays an important role in assessing the cause of SPH and can allow for the identification of SPH (8). However, the definition of gastric varices is often subjective and indistinct without a uniform standard, which brings difficulties and inconsistences to clinical evaluations and scientific research (13). In this long-term follow-up study, we reviewed all CT images and measured maximum diameter of involved veins, which provided an objective evaluation of the extent of varicose veins. Occlusion of splenic vein results in venous hypertension in the collateral pathways, which carry splenic blood to the superior mesenteric and portal veins. It was reported that the collateral pathways mainly occurred through gastroepiploic vein into superior mesenteric vein or short gastric vein through gastric fundus vein, and gastric coronary vein drained into the portal vein (3). Our results were in accordance with the literature. In addition to these common varices, some rarely reported varices were observed, including middle colonic, omentum branch of the gastroepiploic, pancreaticoduodenal, right gastric, left adrenal, and dorsal pancreatic veins, which deserve the attention of clinicians and radiologists, especially for the left adrenal varices. Portal-systemic shunt was rarely observed in patients with SPH due to the low pressure, however, we detected formation of spontaneous gastro-systemic shunt suggested by the enlargement of the left adrenal vein. Most SPHs are asymptomatic and often found incidentally on investigation; thus, their exact time of onset is unknown (14). There was a large difference between different studies in the median time to the detection of SPH, and this time ranged from 12 days to 8 months, which might be a result of different participants and CT detection times among these studies (3, 4). Thus, studying the dynamic changes in gastric varices is very helpful to understand the whole natural history of SPH. In this study, we confirmed that the dynamic changes in gastric varices in SPH patients were associated with a long-term, slowly progressing disease course, and the median time to reach the diagnostic criteria of SPH was 9 weeks. Long-term dynamic monitoring is necessary for the patients with suspicious varices.

Splenic venous thrombosis or occlusion is strongly associated with the occurrence and development of SPH. Varices were identified in 53% of patients with pancreatitis-induced splenic vein thrombosis (15). We also found that the proportion of splenic venous thrombosis was significantly higher in patients with SPH. However, the rate of occlusion or stenosis was even higher than that of thrombosis (75.4% vs. 23.4%). This result indicated that patency of splenic vein was affected not only by thrombosis but also by other factors, such as the stimulation of pancreatin and inflammatory mediators, coagulation dysfunction, and compression of necrotic tissue (12). Additionally, dynamic observations have showed that thrombosis of splenic vein was detected within 1 month after the onset of AP but undetected 3 months after the onset of AP. The condition of undetected thrombosis might be as a result of disappearance of the thrombosis or difficulty on detection by imaging due to the influence of occlusion of veins. Unlike thrombosis, stenosis or occlusion of splenic vein tended to appear at the early stage of the disease and persist during the one-year follow-up period, and the dynamic changes of stenosis or occlusion were more relevant to the changes in SPH, with the proportion of patients diagnosed with SPH increasing over time. Furthermore, multivariable analysis demonstrated that occlusion and stenosis of the splenic vein, but not splenic vein thrombosis, were independent risk factors for SPH.

Investigating risk factors and developing predication models are important steps to distinguish high-risk cases from low-risk cases. It was reported that male sex, local complications, recurrent AP, hypertriglyceridemia, glucose level >10 mmol/L, smoking, and infection of walled-off necrosis were risk factors for SPH (4). Part of our results was basically inconsistent. We found that male sex, high BMI, prolonged PT, and patency of splenic vein were independent risk factors for SPH. The specific reason why SPH was more common in males needs further exploration (3, 4). Obese patients were more prone to have a more serious disease with larger extent of pancreatic necrosis (16). The presence, location, and extent of pancreatic necrosis were strongly related to stenosis or occlusion of splenic vein due to the anatomical location (17). Coagulation dysfunction, which was manifested as prolonged PT, was reported to be a risk factor for splenic vein thrombosis, and the use of anticoagulation therapy is controversial (18, 19). Currently, no studies have reported scores or prediction models for SPH in AP patients. We developed a novel nomogram based on male sex, high BMI, prolonged PT, and the patency of splenic vein and validated that the model could predict SPH with good discrimination, calibration and clinical utility. This is clinically important because it provides a simple model for identifying patients at high risk for SPH at early stage of the disease. Based on this information, a clinician might provide suitable medical consultation, correct judgment on the prognosis and proper advice regarding the follow-up schedule, and clinicians can identify the suspicious cause as soon as possible when gastrointestinal bleeding or other complications happen during the follow-up.

**FIGURE 5:**
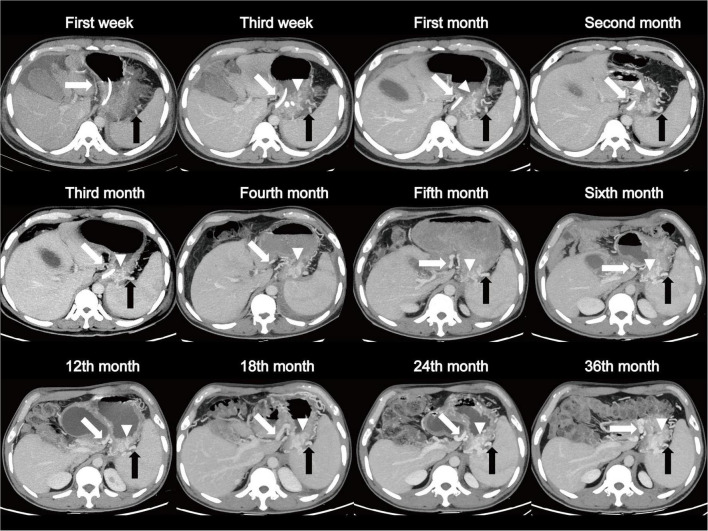
A patient, 39-year-old male, diagnosed as SAP complicated with SPH involving gastric fundus vein (arrow head), short gastric vein (black arrow), and gastric coronary vein (white arrow). Dynamic changes show a long-term, slowly progressing course of SPH over 3 years of follow-up. SAP, severe acute pancreatitis; SPH, sinistral portal hypertension.

Watchful waiting is an acceptable course of management in asymptomatic individuals (8). However, how clinicians should monitor these patients is still an important clinical issue. Herein, we reported a case with regular and frequent follow-up for as long as 3 years. The patient was a 39-year-old male diagnosed with SAP complicated with SPH involving gastric fundus vein, short gastric vein, and gastric coronary vein, as well as stenosis and occlusion of splenic vein (Figure 5 and Supplementary Figure 1). We observed that both SPH and the occlusion of splenic vein had developed slowly and did not resolve over 3 years, but the patient did not have any symptoms, indicating that frequent follow-up might be unnecessary. Because of the following reasons: (1) most SPHs are asymptomatic; (2) dynamic changes in varices are slow and don’t usually recover for a long time; and (3) frequent follow-up and CT increase the patients’ financial burden and medical cost, we advise that the follow-up interval should not be frequent. In total, for patients with MSAP and SAP, especially males and obese patients with abnormal splenic vein and coagulopathies, prolonged and close monitoring with CT examinations every 3 months within the first year of AP onset may be suggested in order to identify SPH as early as possible. For those patients with known SPH, one CT per year may be enough, while more importantly, preventive measures such as appropriate dietary restrictions and patient education for an early recognition of gastrointestinal bleeding should be provided. Further studies are needed to validate the feasibility of the follow-up plan.

This study has several limitations. First, although CT imaging was objective, the results might be affected by the radiologists’ subjective judgments. To reduce the bias, the imaging analysis was conducted by two experienced radiologists who were blinded to other clinical data, and interobserver agreement was considered to be good. It would be more accurate if both magnetic resonance imaging (MRI) and constrast-enhanced CT could be used to assess the imaging features. Second, although the model was derived from our data and internal validation showed good performance, an external validation is still needed. Third, confounding factors were also considered in all observational and retrospective studies. Although we attempted to account and adjust for potential confounding variables, the possibility of unmeasured risk factors still existed.

## Conclusion

We confirmed that the dynamic changes in varices in SPH were long-term, slowly progressing, and we developed a novel nomogram for the early prediction of SPH with good discrimination, calibration and clinical utility. A flexible follow-up plan was suggested in male obese patients with abnormal splenic veins and coagulopathies. Additional prospective studies with larger sample sizes are needed to confirm our findings.

## Data Availability Statement

The raw data supporting the conclusions of this article will be made available by the authors, without undue reservation.

## Ethics Statement

The studies involving human participants were reviewed and approved by Ethics Committee of The First Affiliated Hospital of Nanchang University. Written informed consent was waived due to the retrospective nature of the study.

## Author Contributions

CY reviewed the all CT images. LD designed the study, analyzed the data, and wrote the manuscript. MJ collected the clinical data. CY, LD, and MJ contributed equality to this work and then should be considered co-first authors. QL helped in reviewing CT images. XH, YL, and HK helped in finishing follow-up. HX, WH, and LX participated in its design and helped in analyzing data. XZ revised the manuscript. NL and YZ made substantial contributions to conception, design and coordination of the study. All authors read and approved the final manuscript.

## Conflict of Interest

The authors declare that the research was conducted in the absence of any commercial or financial relationships that could be construed as a potential conflict of interest.

## Publisher’s Note

All claims expressed in this article are solely those of the authors and do not necessarily represent those of their affiliated organizations, or those of the publisher, the editors and the reviewers. Any product that may be evaluated in this article, or claim that may be made by its manufacturer, is not guaranteed or endorsed by the publisher.
