# Supplementary material for: Dynamic Changes and Nomogram Prediction for Sinistral Portal Hypertension in Moderate and Severe Acute Pancreatitis
Source: Front Med (Lausanne). 2022 May 26;9:875263. doi: 10.3389/fmed.2022.875263 (PMC9198833; doi:10.3389/fmed.2022.875263)
Supplement: Supplementary file 1 [file Data_Sheet_1.docx]

*Supplementary Material*

# Supplementary Figure


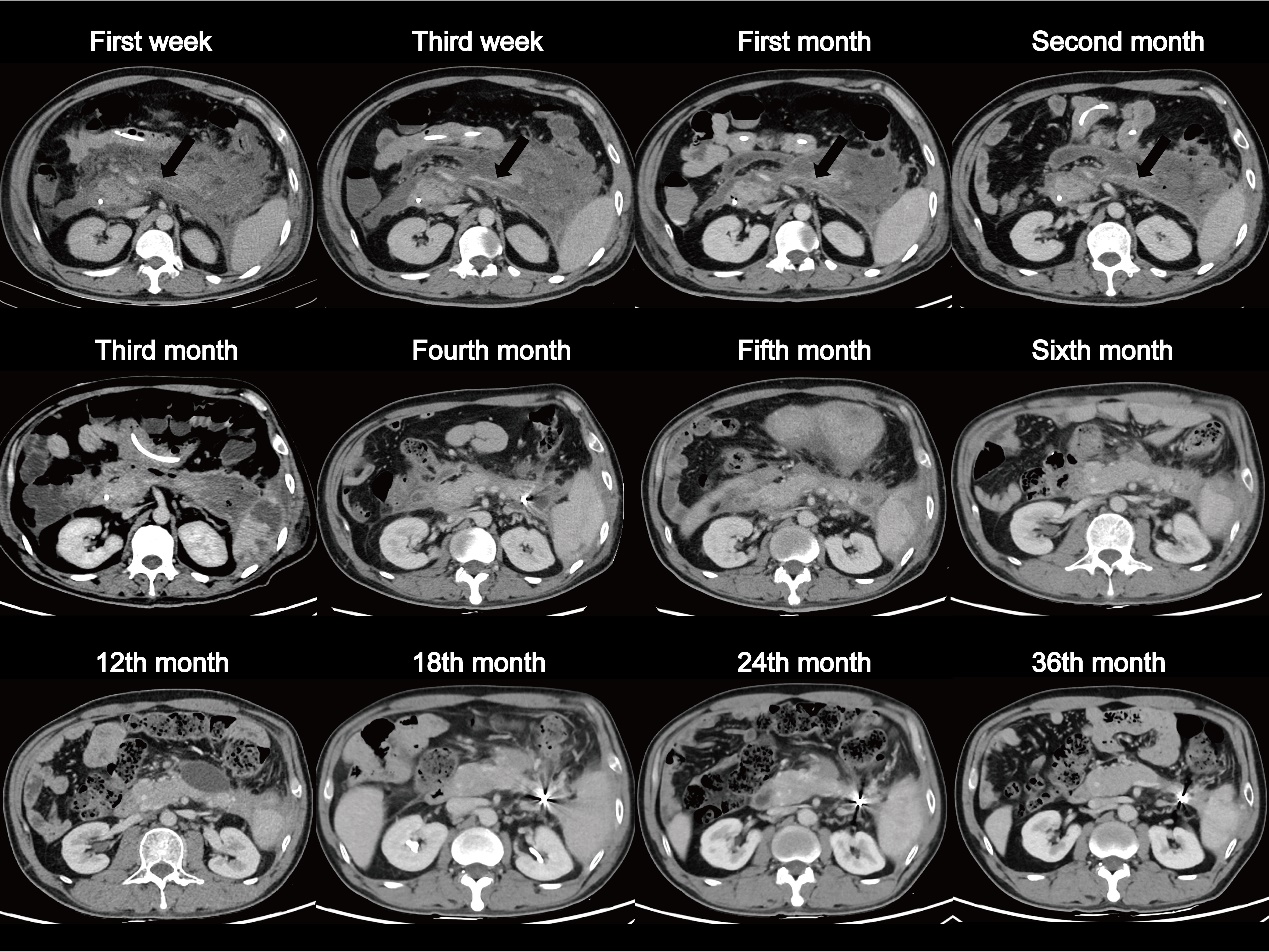
 **Supplementary Figure 1.** A patient, 39-year-old male, diagnosed as SAP complicated with SPH. The dynamic changes of the patency of splenic vein show stenosis of splenic vein within two months of the onset of AP, and persistent occlusion after two months (black arrow). SAP, severe acute pancreatitis; SPH, sinistral portal hypertension; AP, acute pancreatitis.


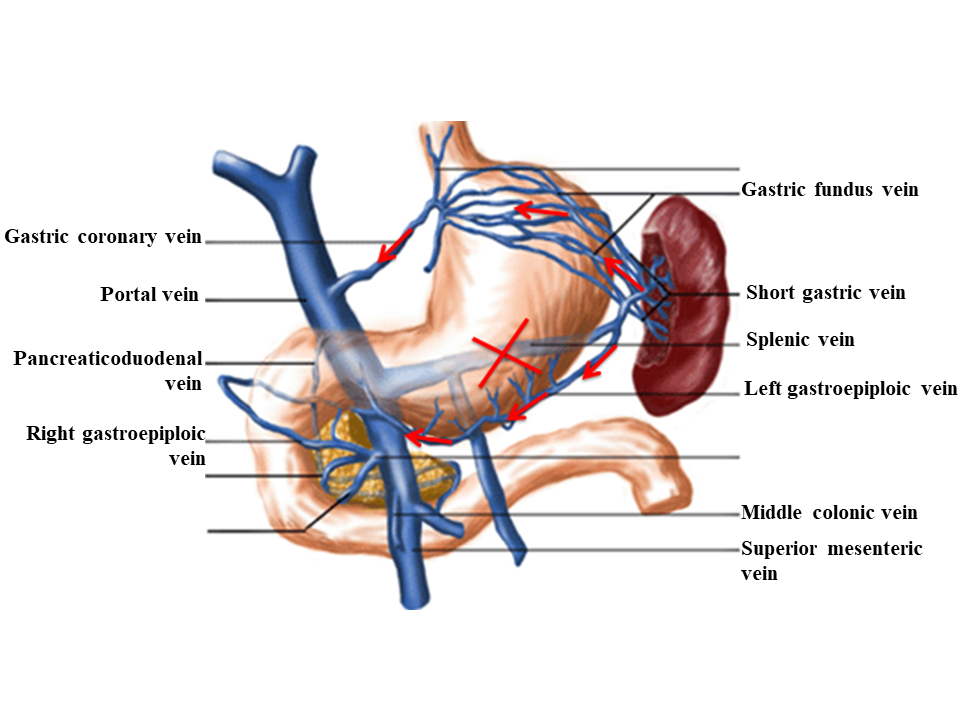


**Supplementary Figure 2.** The main collaterals vessels in sinistral portal hypertension.

# Supplementary Tables

Table S1. The dynamic changes of involved veins in patients with and without SPH*

|  | First week | | | First month | | | Third month | | | Sixth month | | | 12^th^ month | | |
| --- | --- | --- | --- | --- | --- | --- | --- | --- | --- | --- | --- | --- | --- | --- | --- |
|  | SPH | Non-SPH | P value | SPH | Non-SPH | P value | SPH | Non-SPH | P value | SPH | Non-SPH | P value | SPH | Non-SPH | P value |
| Diameter of gastric fundus vein, mm | 0 (0,2) | 0 (0,0) | **<0.001** | 2 (0,3) | 0 (0,0) | **<0.001** | 3 (0,4) | 0 (0,0) | **<0.001** | 3.5 (2,4.5) | 0 (0,0) | **<0.001** | 4 (3,5) | 0 (0,0) | **<0.001** |
| Diameter of short gastric vein, mm | 3 (2,4) | 2 (0,2) | **<0.001** | 3 (2,4) | 2 (0,2) | **<0.001** | 4 (3,5) | 2 (0,2) | **<0.001** | 4 (3.5, 4.5) | 2 (0,2) | **<0.001** | 4 (3,5) | 2 (0,2) | **<0.001** |
| Diameter of gastric coronary vein, mm | 4 (3,5) | 3 (3,4) | **<0.001** | 5 (3.5,6) | 3 (3,4) | **<0.001** | 5 (4,6) | 3 (3,4) | **<0.001** | 6 (4,7) | 3 (3,4) | **<0.001** | 6 (5,7.5) | 3 (3,4) | **<0.001** |
| Diameter of left gastroepiploic vein, mm | 4 (3,5) | 3 (2,3) | **<0.001** | 5 (4,6) | 3 (2,3) | **<0.001** | 5 (4,6) | 3 (2,3) | **<0.001** | 6 (4,7) | 3 (2,3) | **<0.001** | 6 (5,6.5) | 3 (2,3) | **<0.001** |
| Diameter of right gastroepiploic vein, mm | 4 (4,5) | 3 (3,4) | **<0.001** | 6 (4,7) | 3 (3,4) | **<0.001** | 6 (4,7) | 3 (3,4) | **<0.001** | 7 (5,8) | 3 (3,4) | **<0.001** | 6 (4.5,7) | 3 (3,4) | **<0.001** |
| Diameter of middle colonic vein, mm | 3 (1,4) | 3 (2,3) | 0.170 | 4 (2,5) | 3 (2,3) | **0.029** | 4 (2,4.5) | 3 (2,3) | 0.073 | 3.5 (1.5,5) | 3 (2,3) | 0.078 | 4 (3,5) | 3 (2,3) | **0.017** |

Date are median (interquartile ranges). SPH, sinistral portal hypertension. *The maximum diameters of involved varices were recorded during the long-term follow-up. P<0.05 was bolded.

Table S2. The dynamic changes of involved portosplenomesenteric veins in patients with SPH

|  | First week | | First month | | Third month | | Sixth month | | 12^th^ month | |
| --- | --- | --- | --- | --- | --- | --- | --- | --- | --- | --- |
|  | SPH (n=17) | Non-SPH (n=77) | SPH (n=36) | Non-SPH (n=58) | SPH (n=62) | Non-SPH (n=32) | SPH (n=73) | Non-SPH (n=21) | SPH (n=88) | Non-SPH (n=6) |
| Portal vein |  |  |  |  |  |  |  |  |  |  |
| Normal | 14 (82.4%) | 69 (89.6%) | 30 (83.3%) | 52 (89.7%) | 55 (88.7%) | 25 (78.1%) | 61 (83.6%) | 18 (85.7%) | 77 (87.5%) | 5 (83.3%) |
| Stenosis | 3 (17.6%) | 7 (9.1%) | 6 (16.7%) | 5 (8.6%) | 6 (9.7%) | 6 (18.8%) | 10 (13.7%) | 3 (14.3%) | 9 (10.2%) | 1 (16.7%) |
| Occlusion | 0 (0.0%) | 1 (1.3%) | 0 (0.0%) | 1 (1.7%) | 1 (1.6%) | 1 (3.1%) | 2 (2.7%) | 0 (0.0%) | 2 (2.3%) | 0 (0.0%) |
| Thrombosis | 1 (5.9%) | 5 (6.5%) | 1 (2.8%) | 1 (1.7%) | 0 (0.0%) | 0 (0.0%) | 0 (0.0%) | 0 (0.0%) | 0 (0.0%) | 0 (0.0%) |
| Splenic vein |  |  |  |  |  |  |  |  |  |  |
| Normal | 0 (0.0%) | 23 (29.9%) | 3 (8.3%) | 10 (17.2%) | 6 (9.7%) | 7 (21.9%) | 12 (16.4%) | 6 (28.6%) | 18 (20.5%) | 3 (50.0%) |
| Stenosis | 3 (17.6%) | 14 (18.2%) | 5 (13.9%) | 13 (22.4%) | 12 (19.4%) | 6 (18.8%) | 10 (13.7%) | 2 (9.5%) | 9 (10.2%) | 0 (0.0%) |
| Occlusion | 14 (82.4%) | 40 (51.9%) | 28 (77.8%) | 35 (60.3%) | 44 (71.0%) | 19 (59.4%) | 51 (69.9%) | 13 (61.9%) | 61 (69.3%) | 3 (50.0%) |
| Thrombosis | 2 (11.8%) | 20 (26.0%) | 2 (5.6%) | 3 (5.2%) | 0 (0.0%) | 1 (3.1%) | 1 (1.4%) | 0 (0.0%) | 0 (0.0%) | 0 (0.0%) |
| SMV |  |  |  |  |  |  |  |  |  |  |
| Normal | 16 (94.1%) | 72 (93.5%) | 34 (94.4%) | 52 (89.7%) | 51 (82.3%) | 29 (90.6%) | 63 (86.3%) | 19 (90.5%) | 81 (92.0%) | 6 (100%) |
| Stenosis | 1 (5.9%) | 5 (6.5%) | 2 (5.6%) | 6 (10.3%) | 9 (14.5%) | 3 (9.4%) | 8 (11.0%) | 2 (9.5%) | 5 (5.7%) | 0 (0.0%) |
| Occlusion | 0 (0.0%) | 0 (0.0%) | 0 (0.0%) | 0 (0.0%) | 2 (3.2%) | 0 (0.0%) | 2 (2.7%) | 0 (0.0%) | 2 (2.3%) | 0 (0.0%) |
| Thrombosis | 1 (5.9%) | 4 (5.2%) | 0 (0.0%) | 1 (1.7%) | 0 (0.0%) | 0 (0.0%) | 0 (0.0%) | 0 (0.0%) | 0 (0.0%) | 0 (0.0%) |

Date are absolute numbers (proportions). SPH, sinistral portal hypertension; SMV, superior mesenteric vein. P<0.05 was bolded.

Table S3. Univariate Logistic regression analysis for SPH in patients with AP

|  | B | Unadjusted OR (95%CI) | P |
| --- | --- | --- | --- |
| Sex (ref: female) |  |  |  |
| Male | 1.85 | 6.36 (3.01, 13.45) | **<0.001** |
| Age, yr | -0.002 | 1.00 (0.97, 1.02) | 0.877 |
| BMI (ref: <27.5 kg/m^2^) |  |  |  |
| ≥27.5 | 1.11 | 3.04 (1.36, 6.82) | **0.007** |
| HCT, L/L | -0.02 | 0.98 (0.94, 1.02) | 0.377 |
| BUN (ref: <4.8 mmol/L) |  |  |  |
| ≥4.8 | 0.87 | 2.38 (1.30, 4.35) | **0.005** |
| Cr (<57.6 umol/L) |  |  |  |
| ≥57.6 | 1.09 | 2.96 (1.59, 5.50) | **0.001** |
| CRP, mg/L | 0.001 | 1.00 (1.00, 1.00) | 0.255 |
| PCT, ng/ml | 0.007 | 1.01 (0.99, 1.02) | 0.386 |
| PT (ref: <12.6 seconds) |  |  |  |
| ≥12.6 | 1.37 | 3.92 (2.11, 7.30) | **<0.001** |
| APTT, seconds | -0.01 | 1.00 (0.95, 1.03) | 0.525 |
| D-dimer, mg/L FEU | -0.01 | 0.99 (0.95, 1.04) | 0.656 |
| INR | -0.15 | 0.86 (0.52, 1.43) | 0.558 |
| Fibrinogen, g/L | 0.04 | 1.04 (0.90, 1.20) | 0.627 |
| TT, seconds | 0.01 | 1.01 (0.92, 1.12) | 0.836 |
| Use of LMWH (ref: no) |  |  |  |
| Yes | 0.33 | 1.39 (0.76, 2.54) | 0.284 |
| Splenic vein (ref: normal) |  |  |  |
| Stenosis | 2.5 | 12.12 (4.04, 36.39) | **<0.001** |
| Occlusion | 3.31 | 27.50 (11.04, 68.54) | **<0.001** |
| Splenic vein thrombosis (ref: no) |  |  |  |
| Yes | 2.64 | 14.06 (3.20, 61.75) | **<0.001** |
| PPN (ref: no) |  |  |  |
| Yes | 1.77 | 5.85 (3.12, 10.97) | **<0.001** |
| Fluid collection (ref: no) |  |  |  |
| Yes | -0.21 | 0.81 (0.39, 1.69) | 0.574 |
| Number of fluid collection | -0.05 | 0.95 (0.86, 1.06) | 0.369 |

Receiver operating characteristics (ROC) curves were constructed to determine the optimal threshold for predicting clinical outcomes. SPH, sinistral portal hypertension; AP, acute pancreatitis; OR, odds ratio; CI, confidence interval; BMI, body mass index; HCT, hematocrit; BUN, blood urea nitrogen; Cr, creatinine; CRP, C-reactive protein; PCT, procalcitonin; PT, prothrombin time; APTT, activated partial thromboplastin time; INR, International normalized ratio; TT, thrombin time; LMWH, low molecular weight heparin; PPN, pancreatic parenchymal necrosis. P<0.1 was bolded.
